# High-Fiber Belgian Waffles: Quality Characteristics and Consumer Acceptance, Product-Elicited Emotions, and Purchase Intent Evaluated by Millennial Consumers

**DOI:** 10.3390/foods14234064

**Published:** 2025-11-27

**Authors:** Andrea Velasquez, Brandon Freire, Ryan Ardoin, Georgianna Tuuri, Evelyn Watts, Joan M. King, Yupeng Gao, Witoon Prinyawiwatkul

**Affiliations:** 1 School of Nutrition and Food Sciences, Louisiana State University Agricultural Center, Baton Rouge, LA 70803, USAbfreir2@lsu.edu (B.F.); gtuuri@agcenter.lsu.edu (G.T.); egwatts@agcenter.lsu.edu (E.W.); jking@agcenter.lsu.edu (J.M.K.); ygao@agcenter.lsu.edu (Y.G.); 2 Food Processing and Sensory Quality Research Unit, Southern Regional Research Center, United States Department of Agriculture Agricultural Research Service, New Orleans, LA 70124, USA; ryan.ardoin@usda.gov

**Keywords:** waffles, millennials, consumer perception, purchase intent, high fiber

## Abstract

Dietary fiber can help reduce LDL cholesterol and lower the risk of cardiovascular diseases. This study aimed to characterize the physicochemical properties of Belgian waffles formulated with varying soluble dietary fiber levels [<1 g (control), 5 g, 10 g, and 15 g/serving]. Consumer acceptance, product-elicited emotions, and purchase intent (before and after a health-claim statement was presented) were also evaluated. The waffles’ weight loss, color, and texture were measured. Participants (N = 120; 95% millennial consumers) evaluated six sensory attributes using a 9-point hedonic scale; brown color intensity, softness, and chewiness [just-about-right scale]; purchase intent (PI) [yes/no]; and product-elicited emotions [check-all-that-apply]. Overall liking (OL) and PI were evaluated before and after a high-fiber health claim (HFHC) was presented. Data analysis (α = 0.05) included ANOVA, a *t*-test, the McNemar test, Cochran’s Q test, and penalty analysis. Increasing the fiber did not significantly affect waffle aroma, texture, flavor, or OL. Waffles turned a lighter yellow with the two highest fiber addition levels (greater L* and b* values), resulting in decreased color liking. Waffles with 15 g fiber/serving became significantly less cohesive and chewy; however, texture liking did not significantly decrease. After participants read the HFHC, high-fiber samples outperformed the control in OL and PI scores and elicited positive emotions. Added fiber affected color and texture but maintained consumer acceptability.

## 1. Introduction

In recent years, the U.S. population has increased their consumption of ultra-processed foods formulated with various additives and few whole ingredients [1], while decreasing their intake of fruit and vegetables [2]. Excessive consumption of ultra-processed foods can affect the nutritional quality of diets. Rauber et al. [3] reported that increased consumption of ultra-processed food significantly increased the dietary contents of carbohydrates, free sugars, total fats, saturated fats, and sodium, while decreasing the contents of protein and fiber. This dietary shift has been linked to a higher risk of cardiovascular diseases (CVDs) [4,5].

CVDs are the leading cause of death in the United States [6], and their prevalence is projected to rise in the coming years [7]. CVDs are associated with poor nutrition, physical inactivity, and obesity. Other risk factors include high blood cholesterol, high blood pressure, and diabetes mellitus [8]. Individuals with high blood cholesterol levels and low-density lipoprotein (LDL) cholesterol levels of 160 mg/dL or higher possess a greater risk for CVDs [9,10]. Increasing dietary fiber is one means of lowering LDL and overall cholesterol levels [11].

The U.S. Food & Drug Administration (FDA) defines dietary fiber as “non-digestible and insoluble carbohydrates that are intrinsic and intact in plants, which have physiological beneficial effects to human health” [12]. Dietary fiber can come from plant, microbial, or synthetic sources and is broadly categorized into two types: soluble and insoluble. Neither type is digested or absorbed by the body, but soluble fiber partially dissolves in water, while insoluble fiber does not [13]. Increasing the consumption of dietary fiber through the consumption of nutrient-dense foods, including fruits, vegetables, and whole grains, can improve cholesterol levels and has been shown to lower CVD risk [14,15]. In the USA, grain products are the largest source of fiber, providing 32–33% of fiber intake, followed by vegetables, potatoes, and fruits [16]. An adequate intake for dietary fiber is considered to be about 14 g/1000 kcal per day for both adults and children [17]. However, unawareness of the numerous health benefits of dietary fiber, especially among younger adults [18], suggests a need to promote the development of high-fiber products. This promotion is justified by evidence that consumers prefer high-fiber oriented products, which could positively influence their purchasing decisions [16].

In the US food industry, nutrition claims on product labels must adhere to regulatory standards. In the case of fiber, a product can be labeled as a “good source of fiber” if it contains at least 2.5 g of dietary fiber per serving size (10–19% of the daily value), while a “high source of fiber” claim requires 5 g or more of dietary fiber per serving size (≥20% Daily value) [19]. This labeling standard gives consumers information about the nutritional value of the food product to guide health-conscious decision making.

The millennial generation in the U.S., typically defined as those born between 1981 and 1996, account for about 25% of the total US population. This generation possesses substantial spending power [20], and they are often called the next “baby boomers” due to their economic influence [21]. Moreover, they are also the first generation whose lives are extensively integrated with technology and social media [22]. These factors have deeply influenced their food choices and purchasing decisions [23]. Even with access to nutritional information, this generation frequently chooses to eat at fast food restaurants, consume snacks as a meal replacement, and dine out regularly. They also tend to distrust the mass media nutritional recommendations, relying instead on peer opinions and those provided in close social circles [24], which can contribute to unhealthy dietary habits. A study by Okumus [25] identified key influences on millennials’ dietary patterns, including a busy schedule, demand for convenience, social media trends, belonging to different cultures, emotional and physical well-being, and food prices. However, a study by Molinillo et al. [26] reported that this generation was willing to pay more for healthier food options.

Considering the inadequacy in dietary fiber intake caused by current dietary habits of the U.S. population, this study was performed to assess selected physicochemical properties and consumer perception of high-fiber Belgian waffles.

## 2. Materials and Methods

### 2.1. Raw Materials

Belgian waffles were prepared using all-purpose flour (Great Value, Walmart Inc., Bentonville, AR, USA), granulated sugar, whole milk, eggs (Great Value, Walmart Inc., Bentoville, AR, USA), salt (Morton Salt Inc., Chicago, IL, USA), unsalted butter (Land O’Lakes Inc., Arden Hills, MN, USA), Fleischmann’s instant yeast (ACH Food Companies Inc., Mississauga, ON, Canada), and vanilla extract (McCormick & Co., Inc., Hunt Valley, MD, USA). To enhance the fiber content in Belgian waffles, Fibersol-2AG, a soluble and fermentable dietary fiber derived from corn fiber (Fibersol^®^, ADM’s, Clinton, IA, USA), was incorporated at three concentrations (5.40 g, 10.80 g, and 16.20 g per serving) for treatments W5, W10, and W15, respectively. The control (W0) contained <1 g of dietary fiber contributed by all-purpose flour, while the fiber concentrations for W5, W10, and W15 were calculated based on the composition of Fibersol-2AG (92.5% dietary fiber, 4.95% moisture, 10.8 dextrose equivalent). Then, for 5 g/serving size waffles, 5.40 g of the dietary fiber was added (W5); for 10 g/serving, 10.80 g of the dietary fiber was added (W10), and for 15 g/serving, 16.20 g of the dietary fiber was added (W15). Each serving size weighed 73 g, which is comparable to commercial products (70–75 g). Fibersol-2AG meets the definition of dietary fiber for nutrition labeling purposes, as established by the Codex Alimentarius Commission [27].

### 2.2. Waffle Preparation

For the processing of Belgian waffles, the ingredients were weighed and mixed. First, the dry ingredients, including flour, sugar, and yeast, were mixed in a stand mixer (Pro 600™ Series 6, KitchenAid, Whirlpool Corporation, Benton Harbor, MI, USA) at low speed for 60 s. For treatments W5, W10, and W15, dietary fiber was added during the mixing of the dry ingredients. Next, the wet ingredients—eggs, milk, vanilla and butter—were added to the dry ingredients and mixed for 1 min at low speed and then for 4 min at a high speed to form a waffle dough. The dough was covered and allowed to rest for 150 min at room temperature. After resting, 73 g portions of dough were weighed, placed on trays, and stored under refrigeration until cooking. On the day of the consumer test, the waffle dough prepared the night before was brought to room temperature for 60 min before being baked for 3 min using a KRUPS GQ502 Belgian Waffle Maker (KRUPS, Solingen, Germany).

### 2.3. Weight Loss Measurement

The uncooked dough (UCD; about 73 g portions) was weighed in a tared disposable weight boat using an analytical balance (Cole-Palmer Instrument Company, LLC, Vernon Hills, IL, USA). After baking, the waffle was cooled down to room temperature and weighed to obtain the final waffle weight (FWW). The percentage (%) of weight loss was calculated as follows:

(1) Weight loss (%) = [(UCD − FWW)/UCD)] × 100

For each treatment, the weight loss of ten replicates was recorded, averaged, and reported as the percentage of weight loss.

### 2.4. Color Measurement

The surface color of cooked waffles was measured by reflectance using a colorimeter (Baking Meter BC-10, Konica Minolta, Inc., Tokyo, Japan). Ten replicates per treatment were measured after calibrating the instrument with white and zero standards. Color parameters were reported using the CIELAB system: L* (lightness: 0 = black, 100 = white), a* (+a: redness; −a: greenness), and b* (+b: yellowness; b−: blueness). Additionally, the total color difference (∆E) between sample pairs (W0 vs. W5, W5 vs. W10, and W10 vs. W15) and the control (W0) compared to each formulation was calculated using the following equation:

(2) $$∆E=\sqrt{{\left(L_{1}^{*}-L_{2}^{*}\right)}^{2}+{\left(a_{1}^{*}-a_{2}^{*}\right)}^{2}+{\left(b_{1}^{*}-b_{2}^{*}\right)}^{2}},$$

where ∆L ($L_{1}^{*}-L_{2}^{*}$) is the difference in L* (brightness) between two samples, and ∆a ($a_{1}^{*}-a_{2}^{*}$) and ∆b ($b_{1}^{*}-b_{2}^{*}$) are the differences in the color coordinates of a* and b*, respectively [28]. The ∆E (Delta E) is the measure of change in visual perception of two given colors.

### 2.5. Texture Measurement

Texture Profile Analysis (TPA) was conducted on the Belgian waffles using a TA.XTplus Texture Analyzer (Texture Technologies Corporation, Hamilton, MA, USA), with ten replicates per treatment. After the waffles were cooked, they were cut into 3 × 3 × 1.5 cm pieces for analysis. The instrument, equipped with a 30 kg load cell and a 2-inch diameter aluminum cylinder probe, measured hardness (N), adhesiveness (N), cohesiveness (dimensionless), and chewiness (N) at a test speed of 5 mm/s and 50% strain.

### 2.6. Microbiological Analysis of Belgian Waffles

Before the sensory analysis, microbiological testing was performed on the waffles to make sure they were safe for consumption. One sample of each treatment was used for the microbiology test, which included the aerobic plate count (APC), yeast and mold count, and *Escherichia coli*/coliform count (EC). To prepare the sample, 25 g of cooked product for each treatment was placed in a sterile bag (Whirl-Pak, Nasco LLC, Pleasant Prairie, WI, USA), with 25 mL of phosphate-buffered saline (PBS). Samples were homogenized for 60 s using a stomacher (Easy Mix Biomerieux SA, Marcy-l’Étoile, France). Serial dilutions (10^0^ to 10^−3^) in phosphate saline buffer (PBS) were made and plated onto Aerobic Count Plates, *E. coli*/Coliform Count Plates, and Yeast and Mold Count Plates using 3M^TM^ Petrifilm^TM^ (3M^TM^, St. Paul, MN, USA). For APC and EC, the plates were incubated for 48 ± 2 h at 35 ± 2 °C, whereas for yeast and mold, the incubation time was 96 ± 2 h at 25 ± 1 °C. Then, colonies were counted and expressed as log CFU/g. All tests were conducted in duplicates for each treatment.

### 2.7. Consumer Test and Participants

The sensory evaluation and consumer test involving humans was approved by the Louisiana State University Agricultural Center (LSU AgCenter) Institutional Review Board (IRBAG-21-0063). Consumers were recruited by social media/online platforms (e.g., LinkedIn, Facebook) of the LSU AgCenter and from an LSU-Food Innovation Institute consumer database (“TigerTasters,” Baton Rouge, LA, USA) by emails. A total of one hundred twenty (N = 120) male and female students and faculty from Louisiana State University (LSU) between the ages of 19 and 45 years (of which 95% were millennial consumers) participated voluntarily in this study. All the participants met the following criteria: they were at least 18 years old, they did not have any visual impairment or color blindness, they had consumed or were willing to consume waffles, and they had 10 to 15 min of available time to complete the consumer test. The consumer test of the Belgian waffles was conducted at the LSU AgCenter Sensory Laboratory (Baton Rouge, LA, USA). To collect all the panelist information, Qualtrics (XM) Survey Software (Qualtrics, Provo, UT, USA) was used. Panelists were asked to provide their demographic information and indicated if they regularly consumed waffles.

Each piece of freshly baked waffle (about 8 cm in diameter and 1.5 cm thick) was cut into four portions. Each portion was placed in a 2-oz cup labeled with a three-digit blinding code. Each consumer was served with all four samples together (W0, W5, W10, and W15) following a balanced and randomized complete block design. No carriers or adjuncts (e.g., syrup, fruits, or whipped cream) were served. Unsalted crackers and water were served as palate cleansers. Consumers were asked to evaluate the acceptability of six attributes, including overall visual liking (OVL), color, aroma, texture, flavor, and overall liking (OL) using a 9-point hedonic scale (1 = extremely dislike, 5 = neither like nor dislike, and 9 = extremely like). Additionally, brown color and chewiness were measured with a just-about-right (JAR) scale (1 = not brown/chewy enough, 2 = just about right, and 3 = too brown/chewy). Purchase intent was reported on a yes/no scale. Overall liking and purchase intent were measured again after the following health claim was provided: “Dietary fiber has beneficial effects such as lowering bad cholesterol and protecting us against cardiovascular diseases. According to the US FDA, a daily adequate intake of dietary fiber is 14 g for every 1000 calories consumed per day; this product is considered HIGH in dietary fiber (5, 10, and 15 g of dietary fiber per serving sample).” For the control sample, the message was similar, except that it stated, “This product has less than 1 g of dietary fiber.”

Finally, after the immediate taste-testing of each waffle sample, the product-elicited emotions were preliminarily evaluated using a check-all-that-apply (CATA) method based on the 25-term emotional profile developed and validated by Nestrud et al. [29]. Participants were asked to indicate the presence or absence of each emotion evoked by the product. This emotional profile has been used in real-world scenarios, including food evaluations. This profile includes terms presented in alphabetical order: active, adventurous, aggressive, bored, calm, disgusted, enthusiastic, free, good, good-natured, guilty, happy, interested, joyful, loving, mild, nostalgic, pleasant, satisfied, tame, understanding, unsafe (related to health), wild, warm, and worried.

### 2.8. Statistical Analyses

The physicochemical properties of Belgian waffles [weight loss, color (L*, a*, b*), and instrumental texture measurements] were analyzed (α = 0.05) with analysis of variance (ANOVA) to evaluate mean differences among treatments. To compare acceptance across samples, a one-way ANOVA followed by a post-hoc Tukey’s standardized (HSD) range test (α = 0.05) was used for OVL, color, aroma, texture, flavor, overall liking before (OLB), and overall liking after (OLA). To evaluate significant differences between OLB and OLA health claims, a paired *t*-test comparison was performed (α = 0.05). To evaluate results from the just-about-right (JAR) scale, penalty analysis (mean drop) was applied to the brown color and chewiness attributes to examine how both the liking of color and texture scores and overall liking scores were affected when the evaluated attributes were not ideal (i.e., not JAR). The mean drop was calculated as [a mean liking score of the JAR group—a mean liking score of the non-JAR group] [30]. Also, the McNemar test was used to evaluate significant differences in the frequencies between purchase intent before the health claim (PIB) and purchase intent after the health claim (PIA) was provided to the consumers. For statistical analysis of the emotion terms, only emotions with >10% frequency selected by the participants were analyzed using Cochran’s Q test to evaluate differences among selection frequencies (%) (α = 0.05).

## 3. Results and Discussion

### 3.1. Weight Loss

Calculated weight loss measurements for each treatment are presented in Table 1. All samples were 73 g of dough before cooking. After cooking, weight loss across treatments ranged from 10.53% to 14.03%. This weight loss occurred with an increase in temperature, which first vaporizes moisture from the surface and then from the inner areas of the product to reach the equilibrium pressure [31]. In the present study, only treatment W10 presented significant differences compared to W0 (control) and W5, while no significant differences were observed between W10 and W15 (treatments with the highest added fiber). Although soluble fibers are known to bind water and prevent moisture loss [32], the treatments with high fiber addition (W10 and W15) exhibited the highest percentages of weight loss. These findings can be attributed to the complex interaction between fibers, proteins, and water in the dough matrix. While soluble fibers help bind water, they can also disrupt protein functionality due to competitive interaction with water [33]. This interaction can create a more fragile matrix that struggles to retain moisture, potentially leading to increased weight loss despite the water-binding properties of fibers. In contrast, Nassar et al. [34] observed increased water retention but reduced dough stability when adding dietary fiber from orange peel and pulp to make biscuits.

### 3.2. Color Measurements

The addition of the dietary fiber (Fibersol-2Ag) resulted in lighter and more yellow Belgian waffles, as evidenced by higher L* and b* values (Table 1). Treatments W10 and W15, with the highest dietary fiber concentrations, showed significantly higher L* and b* values compared to treatments W0 and W5, respectively. The color of the fiber source may influence the final product color. In this case, the inherent white color in the dietary fiber powder might be the factor contributing to the increase in lightness and yellow hue. Similar effects were noted by Han et al. [35], who found that using resistant maltodextrin (dietary fiber) in corn snack products resulted in a lighter yellow color. Delta E (∆E) values, which quantify perceptible color differences based on human vision thresholds established by Mokrzycki and Tatol [28], are presented in Table 2. The comparison between the control (W0) and the 5 g/serving high-fiber waffle (W5) yielded an ∆E value of 7.72, indicating that the color difference was detectable by the human eye without expertise. This suggests that the color shift from W0 to higher-fiber samples (W10 and W15) would also be visually apparent. Notably, the ∆E between W5 and W10 was 13.7, signifying an easy distinction even by untrained observers. In contrast, the ∆E between W10 and W15 was 2.5, indicating a detectable but less pronounced color difference compared to the W5-W10 pair. Additionally, the ∆E values between the control waffle (W0) and those with increasing dietary fiber concentrations (W5, W10, and W15) were 7.72, 6.64, and 4.69 for W0 vs. W5, W0 vs. W10, and W0 vs. W15, respectively. All the values exceeded the ∆E threshold of 3.5, indicating that all formulations were visually distinct from W0.

### 3.3. Texture Profile Analysis (TPA)

Food texture is an essential sensation that characterizes food and influences consumers’ preferences [36]. Table 3 presents the results for hardness, adhesiveness, cohesiveness, and chewiness. Hardness measures the force required for a pre-determined deformation, while adhesiveness is the work required to overcome the sticky forces between the food sample and the probe [37]. In the present study, the addition of dietary fiber in different concentrations did not impart significant differences either in hardness or adhesiveness among the treatments.

It is known that the type of fiber affects the hardness of cooked products. Several studies have reported an increase in hardness for products with added dietary fiber, which mainly happens when insoluble sources of fiber are used [33,34,35,36,37,38]. For cohesiveness, the treatment with the highest concentration of dietary fiber (W15) had significantly lower cohesiveness compared to the other treatments. This effect is desirable, as high cohesiveness in waffles has been shown to negatively impact consumer acceptance [39]. Similarly, W15 showed significantly reduced chewiness, reflecting lower energy required for swallowing [37].

### 3.4. Microbiological Testing

Microbiological evaluation was performed to test the safety of the product before conducting the consumer study. For APC, the minimum detection level was 20 CFU/g and for EC, yeast, and mold it was 2 CFU/g. No microorganisms were detected for APC, EC, yeast, or mold. Based on these results, the product was determined to be safe, and the consumer test proceeded.

### 3.5. Sensory Analysis

#### 3.5.1. Consumer Acceptance

The results from a total of 120 volunteer panelists were collected (60% female, 40% male). The study population consisted predominantly of millennials (95%), and a substantial majority (80%) reported prior experience consuming Belgian waffles. The results for consumer acceptability and purchase intent are presented in Table 4. The results show that for consumers, the increase in fiber content from <1 g to 15 g fiber/serving did not significantly affect aroma, texture, flavor, or OLB of Belgian waffles across the treatments. However, the addition of dietary fiber had an effect on consumers’ acceptance for OVL, color, OLA, and percentages of PIB and PIA.

Based on the OVL scores in Table 4, consumers assigned higher ratings to W0 and W5 than to W15. For color liking scores, an inverse relationship was observed between added fiber content and color acceptability. As fiber content increased (particularly in W15), color liking scores decreased (Table 4). This pattern and the data recorded for lightness (Table 1) indicate consumer preference for darker waffles (W0 and W5) over their lighter counterparts (W10 and W15). These findings align with Oliveira et al. [40], who similarly reported that consumers favored darker waffles, suggesting the notion that pale coloration is less desirable in waffles.

Regarding aroma and flavor, which are important for consumer acceptance or rejection of a product, no significant differences were found among treatments (Table 4). These results are consistent with those of Almeida et al. [41], who observed no significant effect on aroma acceptance when incorporating various dietary fiber sources into bread formulations. While other sources of fiber can impact product flavor profile, the dietary fiber used in this study demonstrated minimal sensory interference. Therefore, the use of this dietary fiber ingredient was beneficial in maintaining acceptable flavor and aroma of the waffles.

Regarding texture acceptability, no significant differences were found among the treatments even though the instrumental analysis showed significantly lower chewiness and cohesion values for W15 (Table 3). Therefore, the observed physical changes did not significantly affect texture liking scores (Table 4). These findings are in agreement with those of Mellete et al. [42], who reported no significant differences in hedonic rating for texture attributes in a muffin with added dietary fiber from whole wheat grains.

#### 3.5.2. Overall Liking Before (OLB) and After (OLA) High-Fiber Health Claim

Overall liking (OL) was assessed before and after consumers were exposed to the high-fiber health claim. A previous study by Poonnakasem et al. [43] demonstrated that health benefit statements can significantly enhance OL after claim exposure. In the present study, even though the treatments with higher amounts of fiber received the lowest OLB scores, no significant differences were observed among treatments (Table 4). However, after the health claim was presented to the panelists, all treatments with added dietary fiber higher were scored significantly higher compared to the control (W0), which showed a significant decline.

#### 3.5.3. Purchase Intent Before (PIB) and After (PIA) High-Fiber Health Claim

To determine whether consumers were willing to purchase high-fiber Belgian waffles, purchase intent was evaluated before (PIB) and after (PIA) exposure to the health claim. As shown in Table 4, purchase intent for W0 (<1 g/serving size) decreased significantly after the health claim was presented (56.35% PIB vs. 45.24% PIA). In contrast, the consumers (primarily millennials) showed a positive response for high-fiber treatments (W10 and W15). For W10, PIA increased to 69.05% from 57.94% (PIB), while for W15, it increased from 47.62% (PIB) to 61.11% (PIA) after exposure to the health claim (Table 3). These findings are in agreement with a study by Żakowska-Biemans and Kostyra [44], where the consumers indicated that they would pay the highest price for a bread with a high dietary fiber concentration. As the participants consisted predominantly of millennials in this study, these results suggest a particular awareness of dietary fiber as a valued nutritional attribute.

#### 3.5.4. Just-About-Right (JAR) Scores and Penalty Analysis for Color Attribute (Brown Color)

The JAR scale was used to determine the consumers’ perception of brown color intensity of the four different treatments of Belgian waffles (Table 5). A penalty analysis was used to quantify effects of non-optimal sensory attributes from dietary fiber addition on sample acceptability. A penalty should be considered concerning when the percentage of consumers who selected the non-JAR responses was higher than 20% and the mean drop was greater or equal to 2.0 (a high number of consumers say the attribute level is not right, either too much or not enough, with a large impact on overall liking [45]. As shown in Table 5, 53.97% of consumers rated brown-color as “Not enough” on the JAR scale for W15. This treatment also exhibited the highest lightness value (Table 1) and received the lowest liking scores for color and OVL (Table 4). The perception of insufficient browning in waffles was associated with reduced liking scores for both color and OVL. Interestingly, even though W0 received higher OVL and color liking scores, 22.22% of the participants rated its brown color as “Too much”, resulting in a concerning penalty of 2.38 units (on the 9-point scale), as illustrated in Table 5. These results are consistent with the results shown in Table 4, where lighter brown treatments (W10 and W15) had lower liking scores and higher “Not enough” responses, whereas W5 with a darker brown color had the highest liking score and JAR percentage (73.81%).

Therefore, there is a clear relationship between perceived brown color intensity (JAR scale) and color liking scores (9-point hedonic scale). Also, a penalty of concern was found in samples W10 and W15 for color liking in “Not enough” and “Too much”. Consumers reported that W0, W5, and W10 had a concerning penalty when rated “Too much”. As stated earlier, a decrease in color liking was observed with increasing dietary fiber (Table 4).

#### 3.5.5. Just-About-Right (JAR) Scores and Penalty Analysis for Texture Attribute (Chewiness)

As mentioned earlier, the increase in fiber content from <1 g to 15 g fiber/serving did not significantly affect liking scores for texture (Table 4). However, the chewiness was evaluated with a JAR scale to determine if it influenced the liking scores of texture for each treatment. The penalty analysis of chewiness, presented in Table 6, revealed that only treatment W15 (highest dietary fiber concentration) negatively impacted consumer perception. Nearly half of the consumers (45.24%) rated W15 as “Too chewy”, resulting in mean drops of 2.08 for OLB and 2.01 for texture. Although W10 was also perceived as “Too chewy” by 22.22% of consumers with even higher penalties (2.59 for OLB and 2.97 for texture), its impact was less critical, as most consumers (60.32%) still rated it within the JAR range.

#### 3.5.6. Preliminary Emotion Profile of the Consumers (CATA)

**Figure 1:**
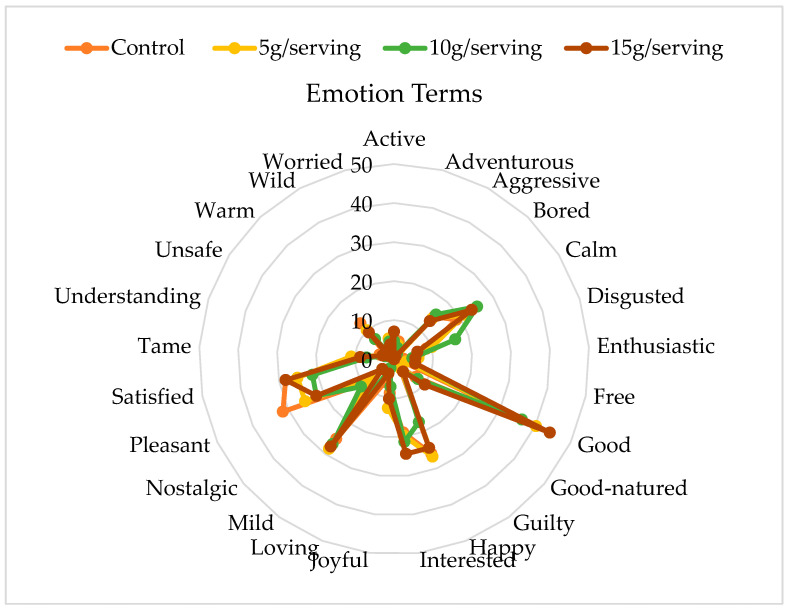
Frequency of emotion terms elicited by the Belgian waffles based on 120 consumer responses.

Food products can evoke positive and negative emotions depending on individual consumer perspectives and product characteristics. As shown in Figure 1, the most frequently selected emotions (reported by ≥20 of participants) were good, satisfied, pleasant, mild, and calm, with the exception of W0 (the control sample), for which “*calm*” was selected by only 18.9% of consumers. Notably, the “good” emotion consistently received the highest frequency compared to the rest of the emotions (Figure 1); this was likely due to consumers associating fiber-enriched products with health and naturalness [44,45]. The positive emotions included good, mild, pleasant, satisfied, and calm, but no statistically significant differences (*p* > 0.05; Cocharan’s Q test) were found among the treatments. In contrast, negative emotions included disgusted, guilty, and bored, but they were selected by fewer participants (just above 5%).

## 4. Conclusions

In this study, the addition of dietary fiber altered the physicochemical properties and influenced consumer acceptability and purchase intent of Belgian waffles. The fiber levels in treatments W5, W10, and W15 were sufficient to claim the waffles as a “good source of fiber”. Results indicate that waffles with higher dietary fiber content (W10 and W15) exhibited a lighter color, which was less acceptable by consumers; this was supported by JAR analysis results, confirming that consumers liked brown color in Belgian waffles rather than a light appearance. Based on the instrumental results for texture, no significant differences in hardness or adhesiveness were found. However, cohesiveness and chewiness were significantly reduced in the treatment with the highest dietary fiber (W15). In the sensory analysis, W0 and W5 showed higher scores for aroma, flavor, and texture; however, no significant differences were found across all the treatments. Notably, an increase in overall liking was observed after providing the high-fiber health claim in all the treatments except in W0 (control). A substantial difference between PIB and PIA was observed between the lowest and the highest fiber concentrations. Certainly, providing a high-fiber health claim increased consumers’ willingness to purchase healthier products even if the added fiber altered some sensory properties. Moreover, from the twenty-five emotions selected, positive emotions were found to be elicited for the high-fiber Belgian waffles.

The findings from this study provided evidence to the food industry that a fiber content claim in compliance with US FDA regulation could positively influence purchase decisions and can serve as an effective marketing tool to promote high-fiber products among millennial consumers without affecting consumer satisfaction. However, a consumer test with a larger sample size should be conducted to validate this finding. Future studies should prioritize investigating alternative fiber sources that can optimize color and texture attributes and evaluating consumer responses, particularly hedonic and emotional appeal, across diverse populations beyond millennials.

## Figures and Tables

**Table 1:** Effects of added fiber on weight loss and color of Belgian waffles.

Treatments	Added Fiber Level	Weight Loss (%)	Lightness (L*)	Redness (a*)	Yellowness (b*)
W0	Control	11.18 ± 1.76 ^B^	60.61 ± 4.79 ^BC^	8.03 ± 1.87 ^A^	14.10 ± 4.34 ^B^
W5	5 g fiber/serving	10.53 ± 1.26 ^B^	56.23 ± 4.59 ^C^	4.28 ± 0.73 ^B^	8.97 ± 3.00 ^C^
W10	10 g fiber/serving	14.03 ± 1.87 ^A^	66.00 ± 3.78 ^A^	7.61 ± 1.97 ^A^	17.96 ± 2.49 ^A^
W15	15 g fiber/serving	12.09 ± 1.66 ^AB^	64.91 ± 4.00 ^AB^	7.70 ± 1.75 ^A^	15.95 ± 2.56 ^AB^

Mean ± standard deviation from ten replications for each treatment. Mean values in the same column followed by different letters are significantly different (*p* < 0.05), based on ANOVA and Tukey’s post hoc standardized (HSD) range test.

**Table 2:** Comparison of total color difference (∆E) values between Belgian waffle samples, as affected by the added fiber.

Treatments	Description	∆E	Interpretation
W0 vs. W5	Control vs. 5 g/serving	7.72	Observer notices two different colors
W5 vs. W10	5 g/serving vs. 10 g/serving	13.7	Observer notices two different colors
W10 vs. W15	10 g/serving vs. 15 g /serving	2.5	Inexperienced observer also notices the difference

According to Mokrzycki and Tatol [28], 0 < ∆E < 1—observers do not notice the difference; 1 < ∆E < 2—only experienced observers can notice the difference; 2 < ∆E < 3.5—inexperienced observers also notice the difference; 3.5 < ∆E < 5—clear difference in color is noticed, 5 < ∆E—observers notice two different colors.

**Table 3:** Effects of added fiber on weight loss and color, and texture profile analysis (TPA) of Belgian waffles.

Treatments	Added Fiber Level	Hardness ^ND^ (Newton)	Adhesiveness ^ND^ (Newton)	Cohesiveness (Dimensionless)	Chewiness (Newton)
W0	Control	0.299 ± 0.031	0.014 ± 0.015	0.519 ± 0.092 ^A^	4.446 ± 0.795 ^A^
W5	5 g fiber/serving	0.291 ± 0.018	0.010 ± 0.013	0.454 ± 0.078 ^A^	3.696 ± 0.433 ^B^
W10	10 g fiber/serving	0.305 ± 0.029	0.0 ± 0.066	0.490 ± 0.066 ^A^	3.500 ± 0.452 ^B^
W15	15 g fiber/serving	0.297 ± 0.029	0.002 ± 0.020	0.331 ± 0.125 ^B^	1.788 ± 0.467 ^C^

Mean ± standard deviation from ten replications for each treatment. Mean values in the same column followed by different letters are significantly different (*p* < 0.05) based on ANOVA and Tukey’s post hoc standardized (HSD) range test. ^ND^: no difference.

**Table 4:** Consumer acceptability scores and purchase intent of Belgian waffles, as affected by added fiber.

Attributes	W0	W5	W10	W15
OVL	7.0 ± 1.65 ^A^	7.09 ± 1.38 ^A^	6.72 ± 1.53 ^AB^	6.42 ± 1.66 ^B^
Color	7.0 ± 1.58 ^AB^	7.13 ± 1.40 ^A^	6.58 ± 1.69 ^BC^	6.21 ± 1.80 ^C^
Aroma ^ND^	6.66 ± 1.84	6.83 ± 1.52	6.51 ± 1.65	6.69 ± 1.64
Texture ^ND^	5.98 ± 1.80	5.99 ± 1.79	5.88 ± 2.10	5.75 ± 2.01
Flavor ^ND^	6.13 ± 1.77	6.3 ± 1.59	6.19 ± 1.88	6.08 ± 1.82
OLB ^ND^	6.13 ± 1.78 *	6.19 ± 1.66	6.09 ± 1.96 *	5.93 ± 1.83 *
OLA	5.66 ± 1.95 ^B,^*	6.48 ± 1.67 ^A^	6.67 ± 1.82 ^A,^*	6.41 ± 1.90 ^A,^*
PIB %	56.35 †	61.11	57.94 †	47.62 †
PIA %	45.24 †	65.08	69.05 †	61.11 †

W0: control sample, 0 g/serving; W5: 5 g/serving size sample, W10: 10 g/serving sample, W15: 15 g/serving sample; OVL: overall visual liking; OLB: overall liking before health claim; OLA: overall liking after health claim; PIB: purchase intent before; PIA purchase intent after (before and after health benefit message was given to consumers; ^ND^: no difference. Means and standard deviations from 120 consumer responses based on a 9-point hedonic scale. Mean values in the same row followed by different letters are significantly different (*p* < 0.05). * Comparing OLB and OLA for each sample, mean values were significantly different according to a paired *t*-test comparison (*p* < 0.05). † Comparing PIB and PIA for each sample, frequency values were significantly different based on McNemar’s exact probability. (*p* < 0.05).

**Table 5:** The JAR frequencies (%) of brown color intensity and penalty analysis * for overall liking (OLB) and color liking, as affected by the non-JAR brown color intensity of Belgian waffles.

		Brown Color			NE (Mean Drop) **		TM (Mean Drop) **	
Treatment	Description	NE (%)	JAR (%)	TM (%)	OLB	Color	OLB	Color
**W0**	Control	6.35	71.43	22.22	1.06	1.92	1.06	2.38
**W5**	5 g fiber/serving	9.52	73.81	16.67	0.88	1.6	0.62	2.39
**W10**	10 g fiber/serving	33.33	55.56	11.11	0.87	2.26	1.45	2.18
**W15**	15 g fiber/serving	53.97	42.86	3.17	0.41	2.14	0.41	0.63

NE = not enough; JAR = just about right; TM = too much; OLB = overall liking before the health claim. * Penalty analysis was performed when the non-JAR frequency (either TM or NE) was ≥20% of consumers. ** The bold values of ≥2.0 indicate “concerning penalties” on liking scores.

**Table 6:** The JAR frequencies (%) of chewiness intensity and penalty analysis * for overall liking (OLB) and texture liking, as affected by the non-JAR chewiness intensity of Belgian waffles.

Treatments	Description	Chewiness			NE (Mean Drop) **		TM (Mean Drop) **	
		NE (%)	JAR (%)	TM (%)	OLB	Texture	OLB	Texture
**W0**	Control	12.9	63.71	23.39	2.48	2.25	1.28	1.28
**W5**	5 g fiber/serving	23.81	53.17	23.02	1.5	1.6	1.65	1.11
**W10**	10 g fiber/serving	17.46	60.32	22.22	2.14	2.22	2.59	2.97
**W15**	15 g fiber/serving	10.32	44.44	45.24	1.47	1.82	2.08	2.01

NE = not enough; JAR = just about right; TM = too much. OLB = overall liking before the health claim. * Penalty analysis was performed when the non-JAR frequency (either TM or NE) is ≥20% of consumers. ** The bold values of ≥2.0 indicate “concerning penalties” on liking scores.

## Data Availability

The original contributions presented in the study are included in the article. Further inquiries can be directed to the corresponding author.
